# Flexibility in MuA Transposase Family Protein Structures: Functional Mapping with Scanning Mutagenesis and Sequence Alignment of Protein Homologues

**DOI:** 10.1371/journal.pone.0037922

**Published:** 2012-05-29

**Authors:** Tiina S. Rasila, Mauno Vihinen, Lars Paulin, Saija Haapa-Paananen, Harri Savilahti

**Affiliations:** 1 Institute of Biotechnology, Viikki Biocenter, University of Helsinki, Helsinki, Finland; 2 Institute of Biomedical Technology, University of Tampere, Tampere, Finland; 3 BioMediTech, Tampere, Finland; 4 Department of Experimental Medical Science, Lund University, Lund, Sweden; 5 Division of Genetics and Physiology, Department of Biology, University of Turku, Turku, Finland; New England Biolabs, Inc., United States of America

## Abstract

MuA transposase protein is a member of the retroviral integrase superfamily (RISF). It catalyzes DNA cleavage and joining reactions via an initial assembly and subsequent structural transitions of a protein-DNA complex, known as the Mu transpososome, ultimately attaching transposon DNA to non-specific target DNA. The transpososome functions as a molecular DNA-modifying machine and has been used in a wide variety of molecular biology and genetics/genomics applications. To analyze structure-function relationships in MuA action, a comprehensive pentapeptide insertion mutagenesis was carried out for the protein. A total of 233 unique insertion variants were generated, and their activity was analyzed using a quantitative *in vivo* DNA transposition assay. The results were then correlated with the known MuA structures, and the data were evaluated with regard to the protein domain function and transpososome development. To complement the analysis with an evolutionary component, a protein sequence alignment was produced for 44 members of MuA family transposases. Altogether, the results pinpointed those regions, in which insertions can be tolerated, and those where insertions are harmful. Most insertions within the subdomains Iγ, IIα, IIβ, and IIIα completely destroyed the transposase function, yet insertions into certain loop/linker regions of these subdomains increased the protein activity. Subdomains Iα and IIIβ were largely insertion-tolerant. The comprehensive structure-function data set will be useful for designing MuA transposase variants with improved properties for biotechnology/genomics applications, and is informative with regard to the function of RISF proteins in general.

## Introduction

Transposable genetic elements constitute a diverse group of discrete DNA segments with a capability of moving within and between genomes [1]. They are abundant in all kingdoms of life and present in virtually every genome examined to date [1], [2]. A wealth of data from sequenced genomes has implicated the fundamental importance of mobile DNA in shaping genomes during evolution [3]–[6]. The increasing knowledge of DNA mobility mechanisms has facilitated the versatile use of transposable elements for research purposes and provided efficient tools for a variety of applications including genome-wide insertional mutagenesis, protein engineering, transgenesis, and gene therapy [7]–[9].

Many mobile DNA elements transpose via a DNA intermediate and are mobilized by an enzyme called transposase. An important class of such transposases shares a structurally and functionally conserved catalytic core domain. This domain folds into a structure first identified in *Escherichia coli* RNase H1 (thus called an RNase H fold), and it includes three catalytically critical acidic amino acids known as the DDE motif [10]–[13]. These DDE-motif transposases belong to a larger group of RNase H fold proteins called a retroviral integrase superfamily (RISF), which also includes retroviral integrases, the Holliday junction resolvase RuvC, the V(D)J recombinase RAG, and Argonaute, the nuclease component of an RNA-induced silencing complex (RISC) [13]–[17]. In addition, the RNase H fold is included in the carboxy-terminal domains of UvrC (DNA-repair) and Prp8 (RNA-processing) proteins, and therefore they are also classified as RISF proteins [13]. Because of a similar molecular architecture, all RISF proteins are expected to use a common mechanism for nucleic acid cleavage and joining reactions [13]. Accordingly, structural and functional insights gained from any member of the RISF proteins can potentially be extrapolated to the entire superfamily.

**Figure 1:**
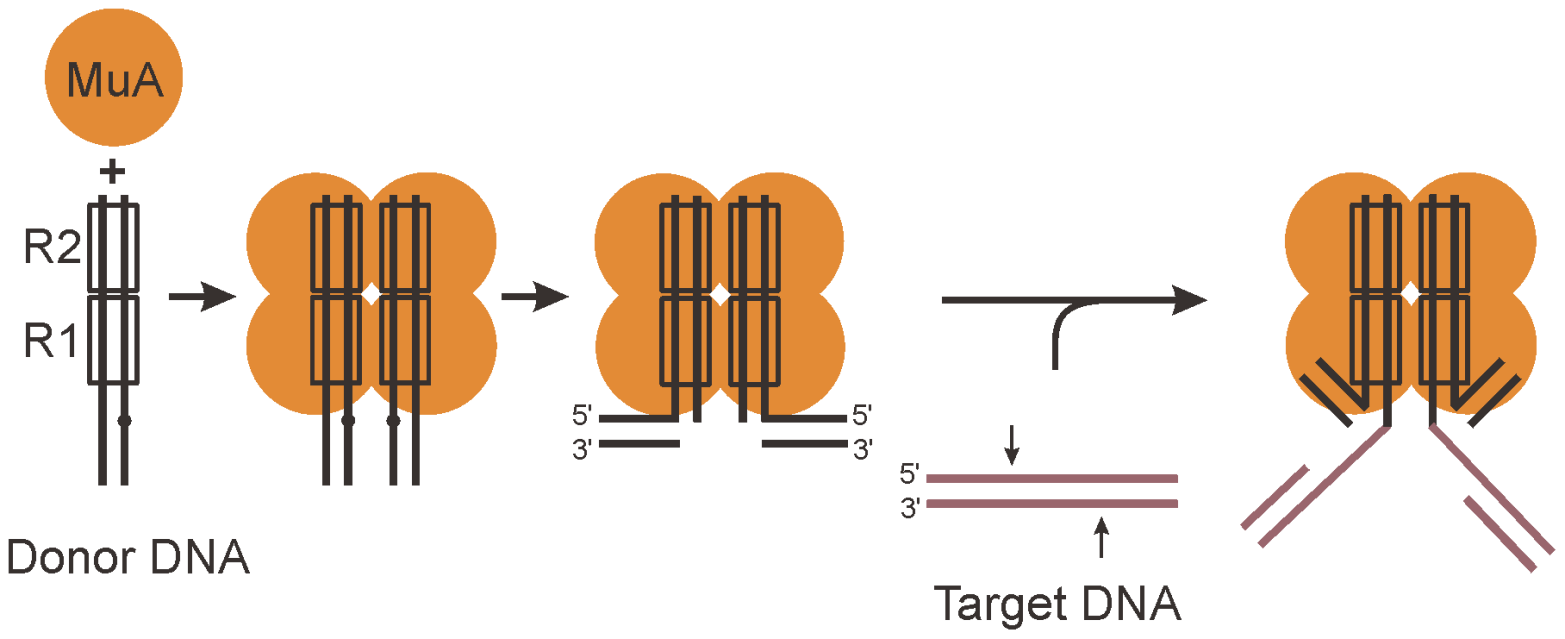
Assembly and function of Mu transpososome core. This pathway is based on *in vitro* studies and utilizes a minimum number of macromolecular components [27]. The *in vivo* assay described earlier [60] and used here (Figure S1) is a close mimic of this minimal-component *in vitro* system with regard the following features: (i) the configuration includes two MuR-ends (with R1 and R2 MuA binding sites), (ii) the phage-encoded MuB protein and (iii) transpositional enhancer are not included. See Figure S1 for a full description of Mu transposition pathway and its comparison to the pathway used in the papillation analysis. The R1 and R2 MuA binding sites are shown as rectangles. MuA is drawn as a tetramer of yellow circles and target DNA is shown in purple. The small arrows on the target DNA indicate the 5-bp staggered locations for strand transfer on the two strands. The dots in the assembled transpososome indicate the Mu end cleavage sites.

Bacteriophage Mu propagates via DNA transposition. Owing to its efficient DNA mobilization capacity *in vivo* [18] and the early development of an *in vitro* system ([19], Figure S1), it has served as an important model system for DNA transposition studies [20]. Mu encodes MuA transposase, a well-characterized member of RISF [12], [13], [21], [22], which catalyzes the critical steps of transposition: (i) initial cleavages at the transposon-host boundaries (donor cleavage) and (ii) covalent integration of the transposon into the target DNA (strand transfer). These steps proceed via sequential structural transitions within a nucleoprotein complex, a transpososome [20], [23], [24], the core of which contains four MuA molecules and two synapsed transposon ends ([25]–[27], Figure 1). *In vivo*, the critical MuA-catalyzed reaction steps also involve the phage-encoded MuB targeting protein, host-encoded DNA architectural proteins (HU and IHF), certain DNA cofactors (MuA binding sites and transpositional enhancer sequence), as well as stringent DNA topology [28]. The critical reaction steps mimicking Mu transposition into external target DNA (Figure 1) can be reconstituted *in vitro* using MuA transposase, 50 bp Mu R-end DNA segments, and target DNA as the only macromolecular components [27], [29]. Such a minimal system has been instrumental for the detailed analyses on the molecular mechanisms of Mu transposition [30]–[32]. A versatile use of the reaction series with custom-designed substrates has generated a wealth of tools for molecular biology applications [33]–[38] and produced novel strategies for genetics/genomics research [39]–[43].

MuA is a 75-kDa protein (663 amino acids) and can be divided into structurally and functionally defined major domains (I, II, III) and subdomains (Iα, Iβ, Iγ; IIα, IIβ; IIIα, IIIβ) [44]–[48]. The N-terminal subdomain Iα promotes transpososome assembly via an initial binding to a specific transpositional enhancer sequence [49], [50]. The specific DNA binding to transposon ends, crucial for the transpososome assembly, is mediated through amino acid residues located in domains Iβ and Iγ [46], [47]. Subdomain IIα contains the critical DDE-motif of acidic residues (D269, D336 and E392), which is involved in the metal ion coordination during the catalysis [51], [52]. Subdomains IIβ and IIIα participate in nonspecific DNA binding, and they appear important during structural transitions [26], [52]. Subdomain IIIα also displays a cryptic endonuclease activity, which is required for the removal of the attached host DNA following the integration of infecting Mu [53], [54]. The C-terminal subdomain IIIβ is responsible for the interaction with the phage-encoded MuB protein, important in targeting transposition into distal target sites [55]–[58]. This subdomain is also important in interacting with the host-encoded ClpX protein, a factor which remodels the transpososome for disassembly [59]. While all MuA subdomains are required for efficient phage Mu transposition inside *Escherichia coli*, the terminal subdomains Iα and IIIβ become dispensable in certain *in vivo* and *in vitro* conditions with appropriately altered DNA substrates and/or suitably modified reaction milieu [60], [61].

Databases classify a number of MuA homologues in a variety of bacteria. Their conserved primary sequence and similar domain structure suggest conservation in their function as well. Although it is not known whether these homologues are currently transpositionally active, aligning their sequences should allow the detection of evolutionarily relevant changes within the MuA family of proteins. It is likely that the obtained data will be applicable to other DDE-motif transposase families as well.

High-resolution structures have been determined for nearly all of the individual subdomains of MuA by NMR [45]–[47] or X-ray crystallography [48]. In addition, a low resolution structure of Mu transpososome has been reconstructed using cryo EM [62]. Very recently, also an X-ray structure of Mu transpososome has been resolved. This informative structure includes target DNA and portrays the transpososome architecture at post-integration stage (P. Rice and S.P. Montaño, personal communication). The solved structures generate a platform for future studies and offer a wealth of detailed information for the architectural interpretation of functional data.

To date, studies on the function of MuA within the Mu transpososome have been limited to deletion analyses as well as mutational analyses of single amino acids. Studies on DNA substrate specificity have complemented the analyses and revealed a degree of flexibility within the architectural requirements [63]–[66]. However, for rational protein engineering and further development of Mu-based technology, more structurally oriented functional studies are needed. Here, we report a comprehensive structure-function analysis to map MuA transposase regions that withstand amino acid insertions versus regions that do not tolerate them. By correlating the activities of insertion mutants with MuA structure, important details of the protein function were revealed. As MuA transposase forms an instrumental component within an expanding tool arsenal for a vast variety of genetics/genomics applications, new data with regard its function are critical.

## Materials and Methods

### Escherichia Coli

#### Strains, reagents, and DNA techniques

DH10B [67] was used as a standard cloning host, and DH5α (Invitrogen) was used for routine plasmid DNA isolation and papillation analysis. Standard bacterial cultures were grown in Luria-Bertani (LB) medium or on LB agar plates [68] supplemented with appropriate antibiotic(s) when required: ampicillin (Ap, 100 µg/ml), kanamycin (Km, 25 µg/ml), chloramphenicol (Cm, 10 µg/ml). Electrocompetent cells for cloning and standard competent cells for papillation analysis were prepared as described in [42] and [69], respectively. Plasmid DNA was prepared using appropriate kits from QIAGEN. Restriction enzymes and DNA modifying enzymes were used as recommended by their supplier (New England Biolabs). Antibiotics and arabinose were from Sigma. Lactose was from BDH and 5-bromo-4-chloro-3-indolyl-β-D-galactopyranoside (Xgal) from AppliChem.

#### Generation of insertion mutant plasmid library

Mutation Generation System (Finnzymes) was used to generate a pool of plasmids for papillation analysis, each plasmid containing a 15-bp insertion within the *MuA* gene. Within the 15-bp insertion, 10 bp (TGCGGCCGCA) is derived from the ends of the transposon used, and 5 bp from the duplicated target DNA at the insertion site [37]. Translation through the insertion is dependent on the reading frame and the sequence at the insertion site (Table S1). In detail, a two-fold scale-up of a standard *in vitro* transposition reaction was performed in a total volume of 40 µl with 200 ng of Entranceposon (M1-Kan^R^) as a donor DNA and 770 ng of plasmid pALH6 [70] as a target DNA. Following incubation at 30°C for 2 h, reaction products were extracted with phenol and subsequently with chloroform, ethanol precipitated, and resuspended in 10 µl of water. Individual aliquots (1 µl) were used to electrotransform DH10B electrocompetent cells as described [42]. Transposon-containing plasmid clones were selected on LB-Ap-Km plates. A total of ∼6.2 × 10^4^ colonies were pooled. Plasmid DNA from the pool was isolated, double-digested with NcoI and EcoRI, and subjected to preparative electrophoresis on a 0.8% SeaPlaque GTG agarose gel in TAE buffer [68]. The 3.1-kb DNA fragment pool, corresponding to transposon insertions into the MuA-encoding DNA segment, was isolated using electroelution [68] and consecutive 1-butanol and chloroform extractions. The DNA was ethanol precipitated, resuspended in TE-buffer (10 mM Tris [pH 7.5], 0.5 mM EDTA), and ligated into NcoI-EcoRI double-digested plasmid pTLH1 [60]. The ligation mixture was electrotransformed into DH10B cells as above, and insert-containing plasmid clones were selected on LB-Km-Cm plates. A total of ∼2.7 × 10^4^ colonies were pooled and grown in LB-Km-Cm medium at 37°C for 2 h, after which plasmid DNA from the pool was isolated. The transposon core sequence was eliminated from the plasmid pool by a cleavage with NotI, followed by preparative electrophoresis on a 0.6% SeaPlaque GTG agarose gel, and isolation of the plasmid backbone as above, and recirculation by ligation at low DNA concentration (∼2 ng/µl). The ligation mixture was electrotransformed into DH10B cells as above, and library clones were selected on LB-Ap-Cm plates. A total of ∼2.6 × 10^4^ colonies were pooled and grown in LB-Ap-Cm medium at 37°C for 2 h. DNA was isolated to yield the final insertion mutation library. For the isolation of individual insertion mutant plasmids, DNA from the mutant library was electrotransformed into DH5α cells. Individual clones were selected on LB-Ap-Cm plates and grown in LB-Ap-Cm medium for DNA isolation.

#### Mapping the insertion sites

The 15 bp insertion sites in isolated plasmids were roughly mapped by an initial screen using either NcoI-NotI or NotI-EcoRI double digestions for 608 clones. On the basis of this initial screen, a total of 331 clones were subjected to DNA sequence analysis to reveal the exact location of the insertion in each individual clone. The following primers were used for the analyses: HSP492 (5′-ATCAGACCGCTTCTGCGTTC), HSP493 (5′-GATTAGCGGATCCTACCTGAC), HSP574 (5′-GCCGGACAAGACCGTAACTTG), and HSP680 (5′-GCAACAGGTGCCAGACATTC). The use of these primers produced partially overlapping sequence reads, together covering the entire *MuA* gene region. DNA sequence determination was performed at the DNA sequencing facility of the Institute of Biotechnology (University of Helsinki) by using the BigDye terminator cycle sequencing kit v. 3.1 and ABI 3130 XL sequencer, both from Applied Biosystems.

#### Papillation assay

MuA insertion variants were assayed for their transpositional activity using an *in vivo* analysis that is based on transposon mobilization [60]. This quantitative assay scores transposition events as blue microcolonies (papillae) growing on otherwise whitish *E. coli* colonies. It takes advantage of a plasmid, which contains a *lacZ*-containing reporter transposon and a cassette for arabinose-inducible MuA expression. Briefly, each insertion mutant plasmid was transformed into standard competent DH5α cells (50 µl), and the cells were plated (∼100 colonies per plate) onto LB agar plates supplemented with 100 µg/ml Ap, 20 µg/ml Cm, 0.05% lactose, 40 µg/ml Xgal, and 0.1% arabinose. The plates were incubated at 30°C for 115 h. For the analysis of each mutant, three representative colonies (diameter ∼5 mm) were photographed using Olympus ColorView II digital camera attached to Olympus SZX12 stereomicroscope equipped with Zeiss KL1500 LCD cold light source. The number of papillae in each colony was then enumerated by using the digital imaging program Image Pro Plus (Media Cybernetics). The data were used to calculate the mean value and standard deviation (SD) for each protein variant. The mean value of MuA wild type activity, 234 papillae, was employed as a reference for 100% activity.

#### Bioinformatics analyses

Multiple sequence alignment was made with ClustalW [71] (for sequences obtained from Pfam [72] for DDE2 family (PF02914) members of RNaseH clan (CL0219). Partial sequences and those with extensive gaps were omitted. The NMR and X-ray structures for isolated domains of MuA protein (codes: 1tns, 2ezk, 2ezh, 1bcm) were from Protein Data Bank (PDB) [73]. Locations of protein secondary structural elements were identified with DSSP [74]. The structures were visualized with UCSF Chimera [75].

## Results

**Figure 2:**
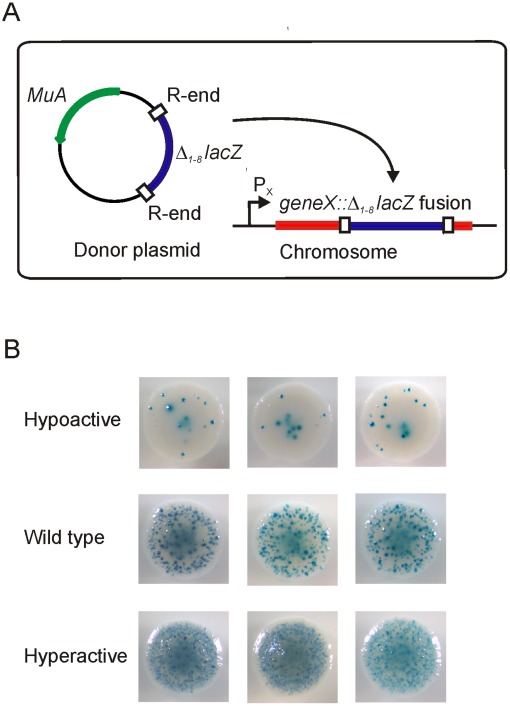
Papillation assay. (A) Phenotypically Lac^–^ *E. coli* strain is transformed with a plasmid carrying a reporter transposon and encoding arabinose-inducible *MuA* transposase gene. Following expression of MuA, the reporter transposon is mobilized. Transposition into an expressed gene (*geneX*) in the correct orientation and reading frame generates a *geneX::lacZ* gene fusion, expressing a protein fusion with a C-terminal β-galactosidase moiety. Such events can be detected as blue papillae in bacterial colonies growing on Xgal-containing indicator plates. This quantitative assay directly measures the activity of the MuA variant analyzed [60]. (B) Colonies from papillation assay. Shown are colonies representing one hypoactive MuA variant (clone #188, Table S1), wild type MuA, and one hyperactive MuA variant (clone #170, Table S1). Three representative colonies per variant are shown, indicating a high degree of reproducibility.

To gain insights into the structure-function relationships of MuA transposase, we generated 233 unique pentapeptide insertion mutants, and these MuA variants were analyzed for function using a quantitative *in vivo* assay (Figure 2). The activity results were correlated with the MuA domain structures, and the data were evaluated with regard to the protein function. In addition, protein sequences of 44 MuA transposase homologues were aligned to reveal telltale signals of evolutionarily succesful insertion/deletion (indel) sites. Combining the quantitative functional data, structural assessment, and alignment-based evaluation generated a comprehensive map of insertion-tolerant versus insertion-intolerant regions in the protein.

### Generation and Characterization of MuA Insertion Mutant Library

**Figure 3:**
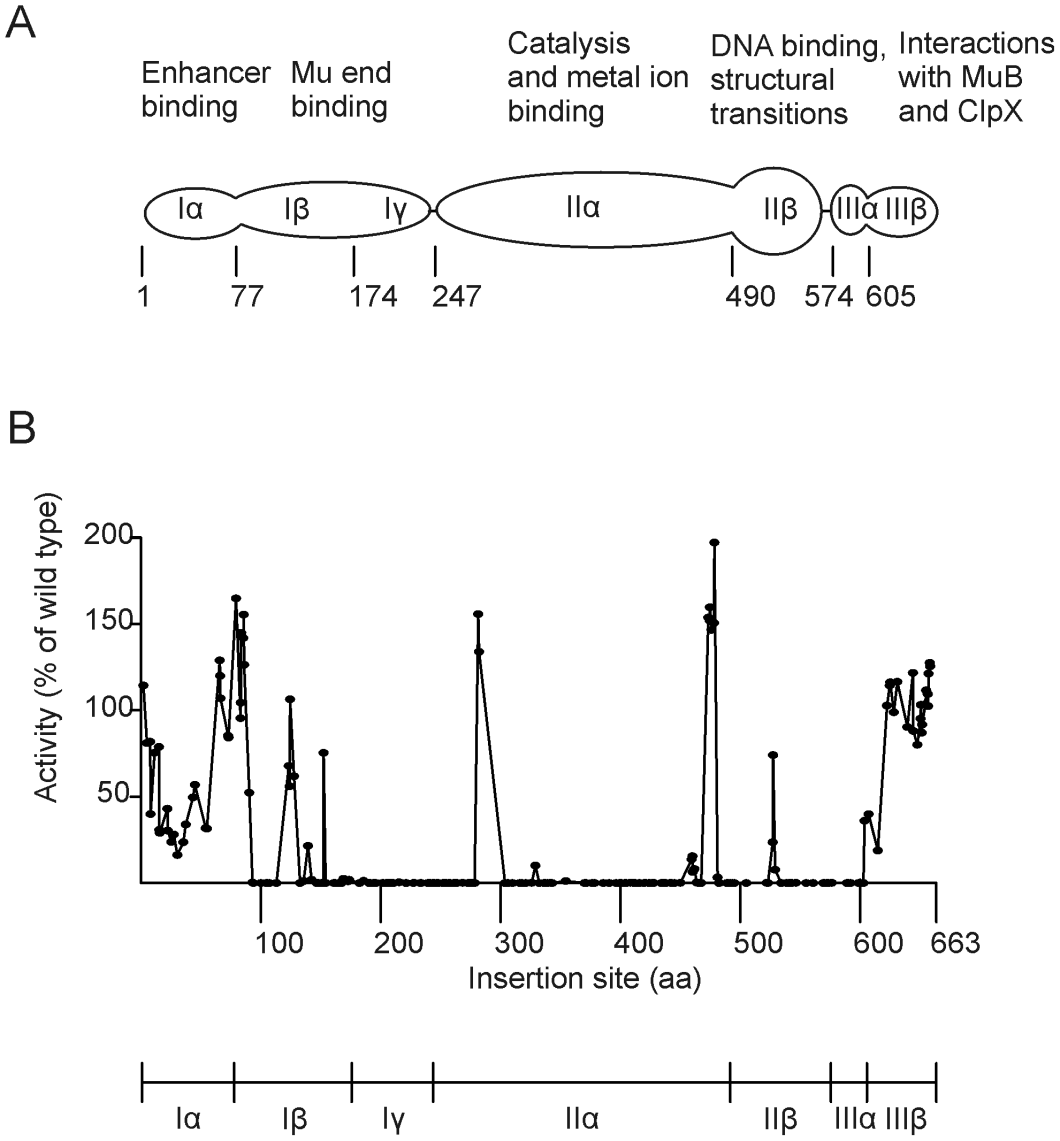
MuA domain structure and location of pentatapeptide insertions with respective transposase activities. (A) Structural organization of MuA with different functions assigned to various subdomains. The numbers correspond to the amino terminus of each subdomain as specified earlier [47]. (B) Enzymatic activities of MuA variants plotted against the 5 aa insertion site of each respective mutant protein. Activities obtained from the papillation assay are presented as a percentage of the wild type activity. For each protein variant the mean from three replicates is shown.

**Figure 4:**
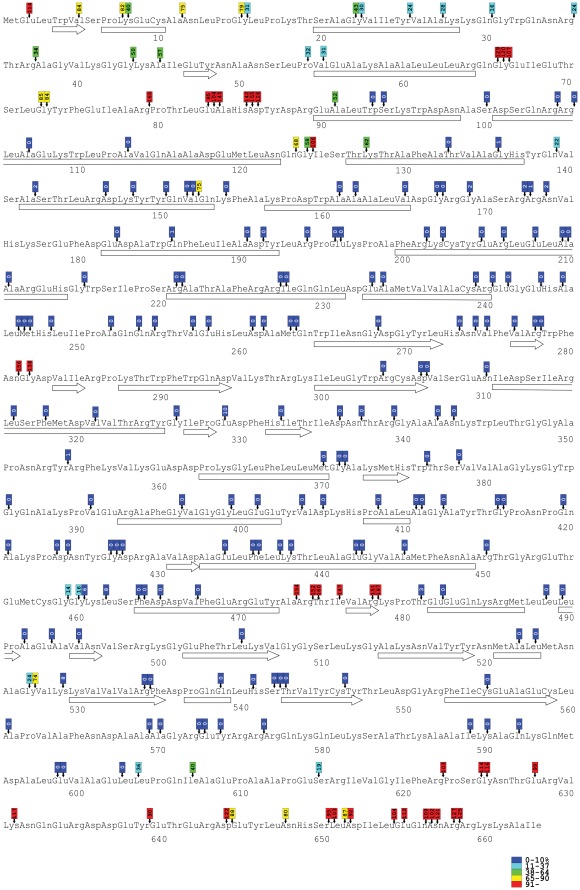
Location of insertions within the amino acid sequence and relative transposition activity of each mutant. Amino acid positions are numbered under the sequence. Secondary structures (determined with DSSP, [74]) are indicated under the corresponding sequence as bars (α-helices) or arrows (β-strands). Small vertical arrows point to the exact location of each insertion (in each three-letter amino acid code, the first letter represents the first nucleotide of the corresponding codon, etc.), and the attached coloured boxes indicate ranges of transposition activity of each mutant relative to the wild type activity (colors denoting the percentage ranges are shown at the lower-right). The number within the boxes indicate the observed relative activity.

We generated a library of plasmids, in which each clone encoded a randomly positioned five amino acid insertion in MuA. The strategy included a final subcloning step, guaranteeing that the insertions were confined solely to the targeted MuA-encoding region. A total of 233 different insertion sites were identified. Their overall distribution as well as their localization within the secondary structural elements of MuA (Figures 3 and 4) was regarded as being sufficient for a reliable structure-function assessment of the protein. The transpositional activity of each of the MuA variants was monitored using a quantitative *in vivo* assay (Materials and Methods, Figure 2). The assay measures transposition frequency as a number of blue papillae appearing on otherwise whitish bacterial colonies. Only fully productive transposition events generate papillae in this assay, meaning that critical alterations at any stage along the transposition pathway will be reflected in the observed transposition frequency. Most of the insertions reduced the activity of MuA (Figures 3 and 4), as a large number of the mutants (187 of 233) exhibited less than 70% of the wild type activity, and more than half of the mutants (125 of 233) were totally inactive with no papillae produced. Many mutants (36 of 233) showed an activity that can be regarded as a wild type or close to the wild type (activity level 70−130%). Some variants (12 of 233) exhibited activity levels exceeding 130%, the highest score being 197%.

### Insertion Tolerance within MuA Domains

MuA is composed of three major protein domains, each containing two or three subdomains (Figure 3A). Our analysis indicated clear differences in insertion tolerance between the subdomains. In particular, the entire subdomain Iα and the main part of subdomain IIIβ tolerated insertions relatively well, as none of the insertions into these subdomains totally abolished the MuA activity. Three of the subdomains, Iβ, IIα and IIβ, tolerated some insertions but only in certain confined regions. Subdomains Iγ and IIIα appeared entirely insertion-intolerant. A five residue insertion forms a substantial change in protein sequence and structure. Accordingly, sites where extra residues can be accommodated indicate regions, which are likely not tightly packed, where the residues do not impede specific structural transitions, and/or regions where sequence conservation is not crucial (e.g. for the enzyme activity, ligand binding or protein folding). In the next subsections, the effects of the insertions are discussed in more detail.

#### Domain I

Subdomain Iα tolerated insertions relatively well. In particular, insertions into the regions forming the subdomain termini (aa 1–15 and 66–76) either retained the wild type activity or reduced the activity only marginally (with one exception: 40% activity at aa 8, frame 3). However, insertions into the subdomain’s central region (aa 16–55) reduced the protein activity noticeably, and only ∼20–60% of the wild type activity was retained. Insertions into the extreme N-terminus of subdomain Iβ (aa 80–86) either retained the wild type activity or substantially increased the activity (up to 165%). Insertions into other regions of subdomain Iβ (aa 91–173) almost invariably abolished the activity entirely. However, within particular helix termini and loop sequences insertions preserved some activity (in one case even up to the wild type level: aa 125, frame 1). Subdomain Iγ was very clearly insertion-intolerant, as the variants had a good coverage of insertions between aa 174–245, and these insertions totally (or in two cases, almost totally) abolished the protein activity.

#### Domain II

In general, subdomains IIα and IIβ were for the most part extremely insertion-intolerant with no papillae formed. Yet, a few non-inactivating insertions were located within these subdomains. Two loop regions tolerated insertions particularly well (aa 282 and aa 474–479). In fact, these insertions increased the protein activity, and the highest activity score (197%) was observed for the insertion at the latter loop. Furthermore, a substantial level of activity (14–74%) was retained with insertions at two additional loop regions (aa 460 and aa 527–528).

#### Domain III

Insertions into subdomain IIIα completely inactivated the protein, except for an insertion into the most C-terminal residue (aa 604, 36% activity). Conversely, subdomain IIIβ tolerated insertions relatively well. While insertions into its extreme N-terminus (aa 607–615) decreased the activity to a relatively low level (19–40%), all other insertions produced essentially wild type activities.

### Structural Assessment

**Figure 5:**
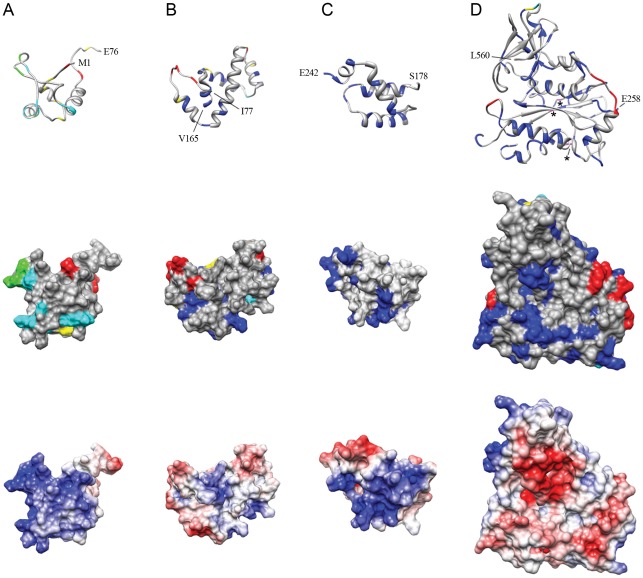
Insertion sites and corresponding transposition activities displayed in the context of the known domain structures of MuA. Shown are ribbon and surface models with the insertion mutant data as well as the electrostatic potential of the wild type protein. (A) Subdomain Iα (aa 1−76, PDB accession code 1TNS). (B) Subdomain Iβ (aa 77−168, 2EZK). (C) Subdomain Iγ (aa 178−242, 2EZH). (D) Domain II (aa 258−560, 1BCO). The first and last residues resolved in the structures are labeled with a corresponding number. The amino acid residues corresponding to each insertion site are coloured according to the MuA activity chart and data defined in Figure 4. The stars in panel D ribbon model highlight the DDE-motif residues shown in magenta within the protein chain.

For the structural assessment of the insertion data, we used the known secondary and tertiary structures of MuA subdomains (Figures 4 and 5). Subdomain Iα involves a winged helix-turn-helix DNA-binding module, which interacts specifically with the Mu transpositional enhancer sequence during the transpososome assembly. As the utilized functional assay does not involve the enhancer [60], insertions into this subdomain were not expected to totally eliminate the protein activity. Fulfilling the predictions, a substantial level of activity was retained even with insertions into secondary structural elements of this subdomain. Subdomains Iβ and Iγ are both involved in the transposon end sequence recognition by MuA. The helices within subdomain Iβ accepted no insertions, except at their extreme termini, consistent with their critical role in DNA binding. One particular region (aa 123–126) between the two helices anchoring the recognition helix accepted insertions without disturbing the protein function. Subdomain Iγ was very clearly entirely insertion-intolerant, as both secondary structural elements and regions between them were not able to accommodate insertions without a total (or almost total) loss of activity. Subdomain IIα forms the catalytic core of MuA. This domain did not accept insertions in any of its secondary structural elements, highlighting the importance of the protein fold integrity for the active site function. However, a few loops between secondary structural elements withstood insertions well, and in certain positions insertions actually increased the protein activity. Subdomain IIβ forms a β-barrel structure, and this structure was highly intolerant of insertions. However, one loop region between secondary structural elements was able to accommodate insertions with only a moderate reduction in the protein activity. As the architecture of the entire subdomain III has not been revealed, we were not able to assess the insertion data with the structure of this subdomain. Nevertheless, it appears that subdomain IIIα is highly intolerant of insertions, whereas subdomain IIIβ withstands insertions well.

### Alignment of MuA Family Members

**Figure 6:**
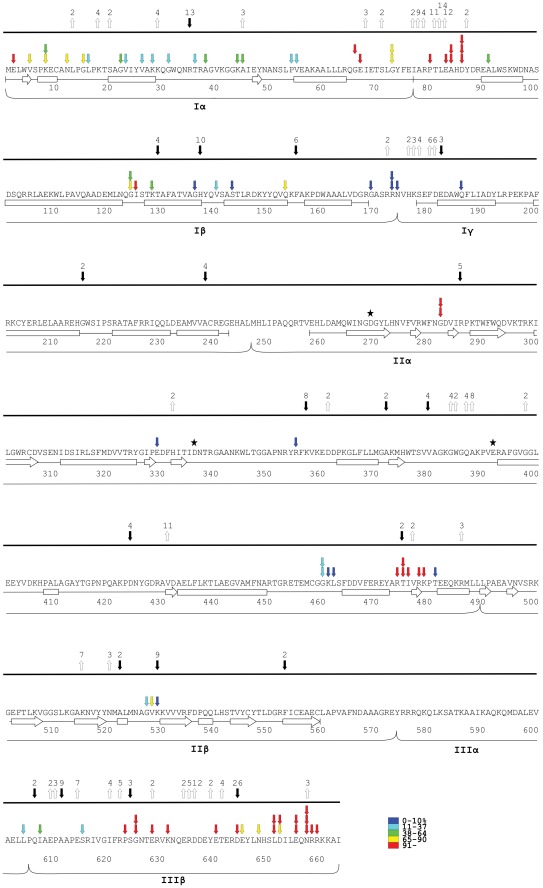
Identification of insertion-tolerant regions in MuA on the basis of pentapeptide insertion analysis and alignment-based data. Above the amino acid sequence are shown with arrows the pentapeptide-insertion tolerant sites (activity level ≥1%) colour-coded as in Figure 4. The percentage range chart is shown at the lower-right. Two or three arrows per site are indicative of insertions involving more than one reading frame. Below the amino acid sequence are shown the secondary structural elements (arrows and rectangles). The elements are connected with line segments indicating the length of each PDB structure. Below the structural elements are shown the subdomains as specified in Figure 3. Above the bolded line, the downward black and upward white arrows represent the alignment-based insertion and deletion (indel) data, respectively (each particular indel precedes the marked amino acid). The maximum indel length at each site is indicated by a number shown above each arrow (data from Figure S1). The stars indicate the DDE-motif residues (D269, D336, E392).

During the evolution of a protein family, insertions and deletions are expected to accumulate in regions not critical for the protein function. To pinpoint such regions in MuA, we aligned the amino acid sequences of 44 MuA transposase family members (Figure S2 and Figure 6). Clear differences were observed among the MuA subdomains with regard the accumulation of insertions and deletions. In particular, massive variation was observed within subdomains Iα and IIIβ. Subdomains Iβ, IIα and IIβ exhibited less variation but nevertheless included relatively long (8–10 aa) insertions. Domain Iγ was even less variable, displaying only a few short (2–4 aa) insertions, and domain IIIα appeared entirely intolerant of insertions and deletions. In general, a vast majority of length variation was confined to regions between the known secondary structural elements of the protein.

### Correlation of Quantitative Functional Data with Sequence Alignment Data

**Figure 7:**
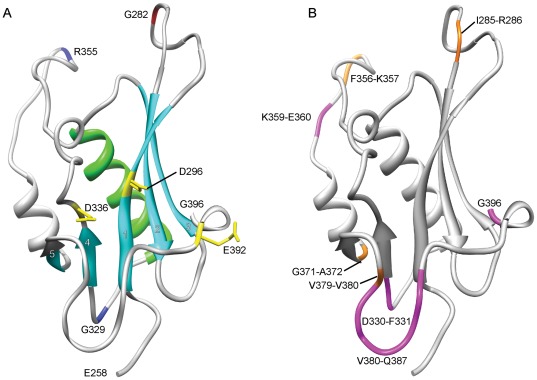
RNaseH fold of MuA encompassing the amino acid region E258–G396. (A) The conserved secondary structural elements, the central β-sheet (with numbered strands 1–5) and the adjacent α-helix, are shown with light blue and light green, respectively. The DDE-motif residues are shown in yellow with exposed sidechains. Pentapeptide-insertion-tolerant sites G282, G329, and R355 are colour-coded as in Figure 4. (B) Mapping of the alignment-based insertions and deletions. The orange-coloured amino acids depict insertion sites, and the accompanying numbers identify the respective amino acids (insertions occur between the specified residues). Maximum length deletions are coloured with magenta and indicated with respective amino acid residue numbers.

We next combined the quantitative functional results and the protein alignment data (Figures 6 and 7). In general, a highly congruent pattern between the two data-sets was evident, although some variation could be discerned. In particular, in both analyses subdomains Iα and IIIβ were extremely tolerant of insertions and deletions, supposedly reflecting a degree of malleability in the functions of these domains as well as their dispensability in assays not involving the IAS or MuB. On the basis of the compiled data, it is clear that the linker sequence between subdomains Iα and Iβ can be highly variable, both in length and composition. It also appears that within the subdomains responsible for Mu end binding, Iβ and Iγ, insertions can be accommodated between secondary structural elements of DNA binding modules. Between subdomains Iβ and Iγ, there appears to be a region withstanding extensive variation. The compiled data from the catalytic core domain, including subdomains IIα and IIβ, indicated four insertion-tolerant regions (roughly: aa 280–290, 350–360, 470–480, and 520–530), as each of these regions were implicated in both of the data sets. Two additional regions of insertion-tolerance were revealed by pentapeptide insertion analysis (around aa 330 and 460). Within the catalytic core, certain sites were implicated in the alignment data set, mostly with alterations of short length. Yet, these regions did not withstand pentapeptide insertions within them or in their close vicinity. As noted earlier, domain IIIα was intolerant of insertions and deletions in both data sets. Overall, the combined results indicated usefulness of the parallel methodologies and generated a comprehensive global map of insertion-tolerant versus insertion-intolerant regions in MuA.

## Discussion

MuA transposase catalyzes the critical DNA cleavage and joining reactions of bacteriophage Mu transposition in the context of a large protein-DNA complex, transpososome, ultimately attaching transposon DNA into target DNA. The overall process is elaborate, including initial DNA-binding and transpososome-assembly phases as well as subsequent structural transitions facilitating catalytic steps. Accordingly, MuA needs to function at several levels of complexity, which arguably is reflected in the modular architecture of the protein. Here, we have analyzed the overall transposition process, and defined those structurally and functionally important regions within the protein that are non-modifiable versus those that are modifiable. The analysis has utilized not only the currently available structural data but also sequence comparisons, providing both architectural and evolutionary perspective. Overall, the analyses conform to earlier studies, portraying MuA as a structurally flexible modular protein. In addition, a high degree of malleability was revealed in certain inter-domain linker regions as well as in loops connecting secondary structural elements within otherwise insertion-intolerant domain core structures.

The *in vivo* transposition assay used scores only fully productive transposition events. Thus, any mutation-inflicted perturbation at any of the steps along the transposition pathway would be reflected in the frequency of emerging papillae. In this study, we used five amino acid insertions as a probe to dissect the function of MuA. In general, such insertions are expected to locally perturb protein structure, pinpointing structurally and functionally important amino acid regions with a high precision, as shown previously in several studies involving many types of proteins [35], [37], [76]–[80]. Indeed, the combined data from all insertion clones allowed a quantitative global assessment of the MuA function. To complement the functional mapping of MuA, we performed an amino acid level comparison of MuA homologues. This comparison of evolutionarily related proteins generated an independent data set paralleling the functional data, but also revealing an extra set of potential insertion-tolerant regions. Combining the results of the two approaches produced a high-definition structure-function map of MuA.

Five amino acid insertions are substantial in size, moving the originally adjacent residues widely apart. In general, such changes are typically not tolerated in the middle of secondary structural elements or in a tightly packed protein core, and changes at or in the vicinity of functionally essential residues are equally harmful. The insertion-tolerant regions in proteins commonly reside on surfaces, at termini of secondary structural elements, in loops connecting secondary structural elements, or in linkers between protein domains. Inside the protein core, insertions are not critical only in cases where the amino acid chain extension does not severely clash with the rest of the protein structure. A particular insertion can modulate the enzyme activity via structural changes affecting catalytic residues or substrate/cofactor binding residues either directly or indirectly through long-range effects. Other possible reasons for the decreased enzyme activity include reduced protein stability and increased propensity for protein aggregation, precipitation, or degradation. All of these phenomena typically involve protein misfolding.

The generated MuA variants displayed a broad variety of activities. While more than half of the variants were totally inactive, some of them exhibited activities substantially exceeding the wild type level (Figure 3B). The inactivating mutations clustered in the globular central domains of MuA (from domain Iβ to IIIα). Considering the above scheme, it is conceivable that many of the inactivating insertions, indeed, critically distorted the overall architecture of each particular domain. This would then lead to a domain inactive in its primary function, which in turn would result in the overall halting of transposition. However, the domains function in concert with DNA and ultimately assemble the catalytically competent tetrameric transpososome. The assembly and catalysis require protein-DNA and protein-protein interactions as well as structural transitions. Accordingly, it is plausible that some of the inactivating mutations perturb these critical transactions, thus acting at a higher-order level.

The non-inactivating mutations were mostly confined to the terminal subdomains Iα and IIIβ, indicating that these subdomains are not critical in the *in vivo* assay used. The data are consistent with our recent MuA deletion variant analysis, indicating dispensability of these subdomains in the same assay [60]. Similar dispensability of subdomains Iα and IIIβ has also been detected *in vitro* [61]. Also certain surface-exposed loop regions within globular domains and interdomain linker regions tolerated insertions well. Most likely, insertions into these loop regions did not distort the global domain structure, and similarly, did not perturb the higher-order transactions. In fact, a substantial increase in the protein activity suggests that these regions may be critical in structural transitions and elasticity offered by the insertions would be beneficial. Similarly, insertions into the linker regions may have increased the flexibility of the protein leading to advancements in higher-order transactions, from assembly to structural transitions during the progression of transpososome development.

Subdomain Iα of MuA is responsible for the IAS binding [49], [50]. While the IAS has traditionally been included in Mu transposition assays, it has been shown that it is dispensable in certain reaction conditions *in vitro* [81]. Furthermore, we have recently deviced an *in vivo* assay, in which the IAS has been omitted intentionally [60], and this assay was used in the current study. Thus, it was expected that the insertions into subdomain Iα would not have had drastic effects on the activity of MuA, and the results were consistent with this prediction. Some level of activity reduction, possibly a result of steric hindrance, could be observed, but nevertheless, none of the insertions into this domain totally abolished the activity. These results are also in concordance with those showing that domain Iα can be deleted from the protein without the loss of activity [60], [61]. The linker region between domains Iα and Iβ (aa 65–89) was highly tolerant of insertions and many insertions actually increased the protein activity. It is plausible that in these cases the transpososome assembly becomes easier, as the insertions into the linker region move the domain Iα further away from the Mu end-binding domain, possibly causing less interference. This is in line with the data showing increased activity when the entire subdomain Iα is absent [60].

**Figure 8:**
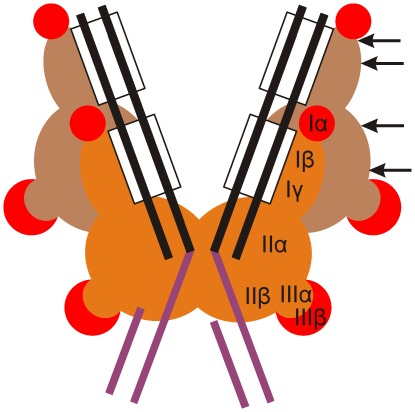
Mu transpososome architecture at post-integration stage and MuA regions allowing pentapeptide insertions. The overall organization is sketched according to the unpublished crystallographic structure of Mu transpososome (P. Rice and S.P. Montaño, personal communication). The structure constitutes an essential framework for a meaningful interpretation of the functional data (see Discussion). Transposon end segments are shown with black lines, MuA binding sites (R1 and R2) are highlighted with rectangles, and the attached target DNA is shown in magenta. The catalytic MuA protomers are shown in orange and the non-catalytic protomers in brown. Insertion-tolerant subdomains are highlighted with red. The arrows (shown only for one protomer) indicate those linker and loop regions, in which insertions are tolerated well (wild type protein activity retained). The catalytic protomers act *in trans*, i.e. the protomer bound to one end catalyzes DNA cleavage and joining reactions in the other end.

Subdomains Iβ and Iγ form the DNA-binding module of MuA, which is involved in the recognition and binding into the MuA binding sites located in the transposon ends. Subdomain Iβ did not tolerate insertions within its DNA-binding surface and in structures anchoring the recognition helices, indicating stringency in the architecture. However, certain regions locating more remotely from the recognition helices tolerated insertions, which indicated flexibility in these structures. Subdomain Iγ is composed of a helix bundle, and according to our analysis its structural requirements are stringent, as insertions were not tolerated at all. When bound to R1 MuA binding site in MuA transpososome, subdomain Iγ makes extensive protein-protein contacts (Figure 8), which may also explain the insertion-intolerance. Insertions between the subdomains of the DNA-binding module caused highly deleterious effects. Recent architectural data from the closest structural homologues of the module (DNA binding domains of Mos1 transposase [82] and CENP-B transposon-derived centromere binding protein [83]) indicate that, in contact with DNA, the homologous linker lies deep in the DNA minor groove. Thus, it is highly likely that the linker in MuA behaves similarly, and certainly our insertion data are consistent with this scenario. Recent structural data from the Mu transpososome, although with a relatively low resolution in the region, also suggest minor groove binding for the linker (P. Rice and S.P. Montaño, personal communication).

The catalytic core of MuA contains an RNase H fold (Figure 7) and includes the critical DDE-motif. In general, the folded structure of the core appears highly stringent in its structural constraints. In our analyses neither pentapeptide insertions nor indels of evolutionary origin were allowed in the secondary structural elements of the fold. The rigidity of the core must reflect the catalytic requirements and perturbations in the architecture are deleterious. Two loop regions in the catalytic core surface region accommodated insertions with ease reflecting their non-critical role in catalysis. Of particular interest is that insertions in these loops substantially (up to twofold) increased the transpositional activity of MuA. The Mu transpososome crystal structure (P. Rice and S.P. Montaño, personal communication) suggests that mutations in the loop region around aa 282 might allow enhanced flexibility between subdomains IIα and IIβ, thus increasing the activity. Further, insertions at aa 474–479 reside in a loop region, which does not make protein-protein or protein-DNA contacts in any of the four MuA protomers within the transpososome. Thus, the activity-enhancing characteristics of insertions in this region may possibly relate to better progression in reaction steps involving structural transitions. Subdomain IIβ folds into a β-barrel structure and is involved in target recognition (Figure 8). In our pentapeptide insertion analysis, this structure appeared rigid, most probably directly reflecting its function in target capture – we observed allowed structural perturbation only in one loop tip (Figure 5D). Consistent with this data, the β-barrel binds target DNA in the Mu transpososome structure, but not with the tips of its loops (P. Rice and S.P. Montaño, personal communication).

Domain III clearly forms two functionally separate units, and this is highlighted also in our study. Subdomain IIIα is a critical determinant with regard to structural transitions. This is clearly apparent in our study as none of the pentapeptide insertions were tolerated, although some activity was retained with insertion to the subdomain’s terminus. In addition, no indels could be discerned in this domain in the evolutionarily relevant sequence comparison. Subdomain IIIβ is involved in MuB and ClpX interactions. As MuB is not involved in the papillation assay used, with this regard the domain should be irrelevant. However, as ClpX is encoded by the host *E. coli* strain used in the current study, we expected to see some variation with insertions into this domain. In line with this prediction, deletion of the entire domain has resulted in substantially reduced activity in the same assay [60]. Contrary to our expectations, insertions into subdomain IIIβ produced essentially wild type level of activities. The reason for this is currently unknown but may relate to alternative transpososome disassembly pathways (recently discussed in [54]).

MuA functions in the transpososome core as a tetramer synapsing two transposon ends (Figure 8). The protomers within the transpososome are functionally and structurally not equal. Although all of the protomers have a structural role, only two of them function in catalysis. This architecture is based on the initial assembly, during which two of the protomers bind to R1 sites and the other two to R2 sites, and the same arrangement is maintained throughout the transpososome development. Very recent data indicate that the target DNA is contacted only through the residues located in the catalytic protomers (Figure 8), indicating that target capture occurs catalytic-protomer-proximally. The tetrameric nature and non-equal contribution of protomers have consequences, as the tolerated five amino acid insertions need to be tolerated in each of the four protomers within the tetramer. Which of the protomers are critical with regard to a particular insertion is an interesting question and a subject of future work.

The current study has revealed important determinants with regard to the function of the Mu transpososome. Accordingly, better transposon tools should be possible to design with the aid of the data. We can envision larger additions, even entire domains, into loops that tolerated insertions. In addition, it is plausible that the linker sequences between domains might be modified to yield transposases with enhanced performance characteristics.

## Supporting Information

Figure S1 A schematic view on the mechanism of Mu DNA transposition. (A) The reaction pathway involving supercoiled plasmid substrates as revealed by a number of *in vitro* studies (reviewed in [28], [84]). This pathway recapitulates the essential features of Mu transposition *in vivo*. It involves a supercoiled plasmid carrying Mu left and right end sequences. The left end and right end are in inverted relative orientation, and each end contains three MuA binding sites (L1, L2 and L3 in the left; R1, R2 and R3 in the right). In addition, the transpositional enhancer (E) is included in the plasmid. The transpososome assembly is facilitated via a transient protein-DNA complex LER (gray oval) involving MuA, Mu end sequences and the enhancer. Once assembled, the transpososome (gray circle) function involves helix opening, transposon end cleavage, target capture, and strand transfer. Subsequently, the transpososome is disassembled via action of ClpX. The resulting transposition intermediate is then processed (repaired or replicated) by the use of host functions. (B) The presumed reaction pathway employed by the *in vivo* papillation assay in this study. In comparison to the pathway described in (A), the papillation assay pathway involves only Mu right end sequences (containing R1 and R2 MuA binding sites), and it does not employ transpositional enhancer sequence and MuB protein. Consequently, the pathway does not involve the LER complex, and overall the pathway is less convoluted.
(TIF)

Figure S2 Alignment of 44 MuA family of transposase proteins.
(XLSX)

Table S1 Compilation of data on MuA variants analyzed.
(PDF)
