# Supplementary figures and images for: Flexibility in MuA Transposase Family Protein Structures: Functional Mapping with Scanning Mutagenesis and Sequence Alignment of Protein Homologues
Source: PLoS One. 2012 May 29;7(5):e37922. doi: 10.1371/journal.pone.0037922 (PMC3362531; doi:10.1371/journal.pone.0037922)

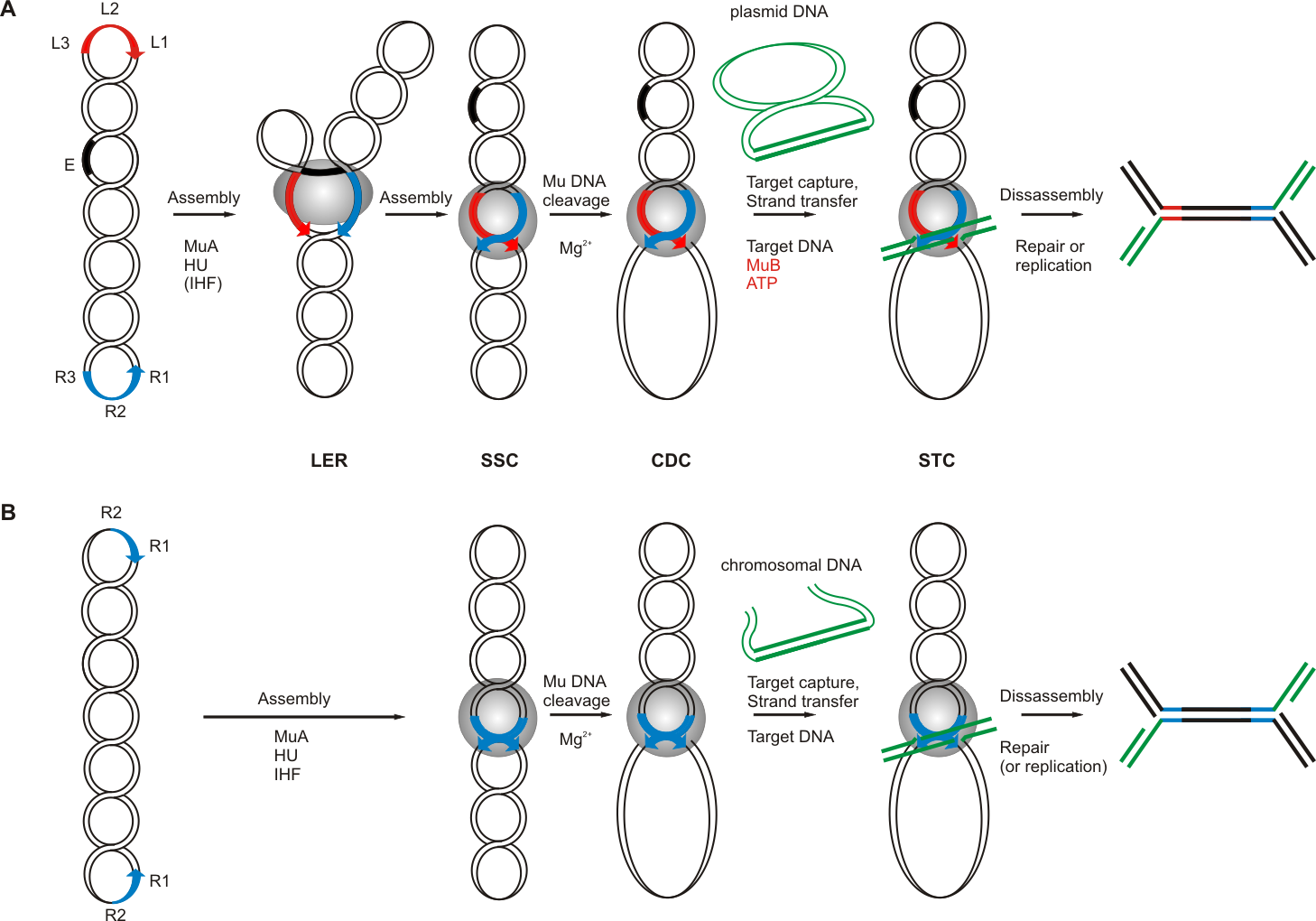

Supplement: Figure S1 — A schematic view on the mechanism of Mu DNA transposition. (A) The reaction pathway involving supercoiled plasmid substrates as revealed by a number of in vitro studies (reviewed in [28], [84]). This pathway recapitulates the essential features of Mu transposition in vivo. It involves a supercoiled plasmid carrying Mu left and right end sequences. The left end and right end are in inverted relative orientation, and each end contains three MuA binding sites (L1, L2 and L3 in the left; R1, R2 and R3 in the right). In addition, the transpositional enhancer (E) is included in the plasmid. The transpososome assembly is facilitated via a transient protein-DNA complex LER (gray oval) involving MuA, Mu end sequences and the enhancer. Once assembled, the transpososome (gray circle) function involves helix opening, transposon end cleavage, target capture, and strand transfer. Subsequently, the transpososome is disassembled via action of ClpX. The resulting transposition intermediate is then processed (repaired or replicated) by the use of host functions. (B) The presumed reaction pathway employed by the in vivo papillation assay in this study. In comparison to the pathway described in (A), the papillation assay pathway involves only Mu right end sequences (containing R1 and R2 MuA binding sites), and it does not employ transpositional enhancer sequence and MuB protein. Consequently, the pathway does not involve the LER complex, and overall the pathway is less convoluted. (TIF) [file pone.0037922.s001.tif]
