# Supplementary material for: Flexibility in MuA Transposase Family Protein Structures: Functional Mapping with Scanning Mutagenesis and Sequence Alignment of Protein Homologues
Source: PLoS One. 2012 May 29;7(5):e37922. doi: 10.1371/journal.pone.0037922 (PMC3362531; doi:10.1371/journal.pone.0037922)
Supplement: Table S1 — Compilation of data on MuA variants analyzed. (PDF) [file pone.0037922.s003.pdf]

Table S1. Compilation of data on MuA variants analyzed[a].

Clone	Insertion site: nucleotide	Insertion site: amino acid (frame)	Insertion translation	Number of papillae[b]	Activity (% of wt)
1	6	2 (3)	DAAAM	268 ± 26	114
2	15	5 (3)	VAAAW	190 ± 27	81
3	23	8 (2)	MRPQP	191 ± 44	82
4	24	8 (3)	NAAAP	94 ± 15	40
5	34	12 (1)	CGRSA	177 ± 27	75
6	45	15 (3)	GAAAP	185 ± 14	79
7	46	16 (1)	CGRTG	72 ± 3	31
8	66	22 (3)	GAAAA	101 ± 15	43
9	67	23 (1)	CGRTG	71 ± 11	30
10	76	26 (1)	CGRIY	56 ± 6	24
11	82	28 (1)	CGRIA	66 ± 19	28
12	91	31 (1)	CGRKQ	38 ± 6	16
13	106	36 (1)	CGRNR	55 ± 10	24
14	112	38 (1)	CGRTR	79 ± 11	34
15	130	44 (1)	CGRSG	116 ± 4	50
16	135	45 (3)	AAAAK	133 ± 17	57
17	162	54 (3)	PAAAL	74 ± 5	32
18	165	55 (3)	VAAAP	74 ± 5	31
19	197	66 (2)	VRPQQ	302 ± 11	129
20	198	66 (3)	GAAAQ	281 ± 30	120
21	199	67 (1)	CGRKG	250 ± 6	107
22	218	73 (2)	VRPHL	200 ± 27	85
23	219	73 (3)	GAAAL	197 ± 8	84
24	238	80 (1)	CGRTR	386 ± 14	165
25	249	83 (3)	DAAAL	223 ± 8	95
26	250	84 (1)	CGRME	244 ± 10	104
27	251	84 (2)	VRPQE	339 ± 10	145
28	256	86 (1)	CGRTH	332 ± 57	142
29	257	86 (2)	VRPHH	364 ± 21	155
30	258	86 (3)	DAAAH	296 ± 30	126
31	272	91 (2)	VRPHE	122 ± 16	52
32	279	93 (3)	CAAAL	1 ± 1	0
33	281	94 (2)	MRPQW	0 ± 0	0
34	300	100 (3)	SAAAA	0 ± 0	0
35	313	105 (1)	CGRKR	0 ± 0	0
36	316	106 (1)	CGRSR	0 ± 0	0
37	321	107 (3)	AAAAL	0 ± 0	0
38	339	113 (3)	AAAAP	0 ± 0	0
39	370	124 (1)	CGRNQ	159 ± 11	68
40	372	124 (3)	GAAAQ	131 ± 7	56

41	373	125 (1)	CGRKG	249 ± 5	106
42	383	128 (2)	MRPQT	145 ± 14	62
43	398	133 (2)	MRPHA	0 ± 0	0
44	407	136 (2)	VRPHA	3 ± 1	1
45	418	140 (1)	CGRNQ	50 ± 7	22
46	427	143 (1)	CGRSA	4 ± 2	2
47	440	147 (2)	VRPQR	0 ± 0	0
48	445	149 (1)	CGRNK	0 ± 0	0
49	451	151 (1)	CGRNY	0 ± 0	0
50	455	152 (2)	VRPHQ	0 ± 0	0
51	456	152 (3)	VAAAQ	1 ± 1	0
52	457	153 (1)	CGRKV	176 ± 10	75
53	462	154 (3)	NAAAQ	0 ± 0	0
54	483	161 (3)	RAAAL	0 ± 0	0
55	486	162 (3)	AAAAA	0 ± 0	0
56	495	165 (3)	VAAAL	0 ± 0	0
57	501	167 (3)	GAAAG	0 ± 0	0
58	502	168 (1)	CGRNG	0 ± 0	0
59	507	169 (3)	GAAAR	6 ± 1	2
60	517	173 (1)	CGRTR	4 ± 1	2
61	518	173 (2)	LRPHR	2 ± 2	1
62	521	174 (2)	MRPHA	4 ± 3	2
63	547	183 (1)	CGRTE	0 ± 0	0
64	557	186 (2)	LRPHW	3 ± 2	1
65	571	191 (1)	CGRIA	0 ± 0	0
66	574	192 (1)	CGRTD	0 ± 0	0
67	582	194 (3)	RAAAL	0 ± 0	0
68	587	196 (2)	LRPQR	0 ± 0	0
69	588	196 (3)	DAAAP	0 ± 0	0
70	604	202 (1)	CGRIR	0 ± 0	0
71	607	203 (1)	CGRSK	0 ± 0	0
72	614	205 (2)	VRPHY	0 ± 0	0
73	618	206 (3)	RAAAE	0 ± 0	0
74	624	208 (3)	DAAAL	0 ± 1	0
75	629	210 (2)	VRPQL	0 ± 0	0
76	630	210 (3)	AAAAL	0 ± 0	0
77	632	211 (2)	VRPQA	0 ± 0	0
78	646	216 (1)	CGRNG	1 ± 0	0
79	663	221 (3)	RAAAS	0 ± 0	0
80	664	222 (1)	CGRTR	0 ± 0	0
81	682	228 (1)	CGRSR	0 ± 0	0
82	683	228 (2)	MRPHR	0 ± 1	0
83	699	233 (3)	DAAAD	0 ± 0	0
84	701	234 (2)	VRPHE	0 ± 0	0
85	718	240 (1)	CGRTC	0 ± 0	0

86	721	241 (1)	CGRSR	0 ± 1	0
87	724	242 (1)	CGRSE	0 ± 0	0
88	730	244 (1)	CGRSE	0 ± 0	0
89	734	245 (2)	VRPQH	0 ± 0	0
90	739	247 (1)	CGRTL	0 ± 0	0
91	740	247 (2)	MRPQL	0 ± 0	0
92	741	247 (3)	IAAAL	0 ± 0	0
93	745	249 (1)	CGRMH	0 ± 0	0
94	756	252 (3)	AAAAP	0 ± 0	0
95	761	254 (2)	LRPQQ	0 ± 0	0
96	764	255 (2)	LRPQQ	0 ± 0	0
97	771	257 (3)	VAAAT	0 ± 0	0
98	774	258 (3)	DAAAV	0 ± 0	0
99	782	261 (2)	VRPHL	0 ± 0	0
100	784	262 (1)	CGRMD	0 ± 0	0
101	789	263 (3)	IAAAA	0 ± 1	0
102	790	264 (1)	CGRTM	0 ± 0	0
103	805	269 (1)	CGRNG	0 ± 1	0
104	819	273 (3)	HAAAL	0 ± 0	0
105	823	274 (1)	CGRNN	0 ± 0	0
106	826	275 (1)	CGRNV	0 ± 0	0
107	830	277 (2)	VRPHF	0 ± 1	0
108	834	278 (3)	RAAAV	0 ± 0	0
109	835	279 (1)	CGRIR	0 ± 0	0
110	844	282 (1)	CGRIN	364 ± 28	156
111	846	282 (3)	GAAAN	313 ± 40	134
112	911	305 (2)	LRPHW	0 ± 0	0
113	918	306 (3)	DAAAC	0 ± 0	0
114	919	307 (1)	CGRSD	0 ± 0	0
115	930	310 (3)	NAAAE	0 ± 0	0
116	948	316 (3)	LAAAR	0 ± 0	0
117	954	318 (3)	FAAAS	0 ± 0	0
118	963	321 (3)	VAAAD	0 ± 0	0
119	978	326 (3)	GAAAY	0 ± 0	0
120	987	329 (3)	DAAAP	24 ± 4	10
121	997	333 (1)	CGRIH	0 ± 0	0
122	1008	336 (3)	DAAAI	0 ± 0	0
123	1012	338 (1)	CGRNN	0 ± 0	0
124	1018	340 (1)	CGRTR	0 ± 0	0
125	1026	342 (3)	AAAAA	0 ± 1	0
126	1029	343 (3)	AAAAA	0 ± 0	0
127	1063	355 (1)	CGRSY	3 ± 1	1
128	1110	370 (3)	IAAAL	0 ± 0	0
129	1113	371 (3)	GAAAM	0 ± 0	0
130	1114	372 (1)	CGRMG	0 ± 0	0

131	1129	377 (1)	CGRNW	0 ± 0	0
132	1135	379 (1)	CGRTS	0 ± 0	0
133	1157	386 (2)	VRPHW	0 ± 0	0
134	1172	391 (2)	VRPQP	0 ± 0	0
135	1189	397 (1)	CGRIG	0 ± 0	0
136	1198	400 (1)	CGRSG	0 ± 0	0
137	1203	401 (3)	DAAAL	0 ± 0	0
138	1211	404 (2)	VRPQY	0 ± 0	0
139	1215	405 (3)	DAAAV	0 ± 0	0
140	1225	409 (1)	CGRNP	0 ± 1	0
141	1232	411 (2)	VRPQL	0 ± 1	0
142	1233	411 (3)	AAAAL	0 ± 0	0
143	1237	413 (1)	CGRTG	0 ± 0	0
144	1247	416 (2)	VRPHT	0 ± 0	0
145	1248	416 (3)	GAAAT	0 ± 0	0
146	1259	420 (2)	LRPHP	0 ± 0	0
147	1262	421 (2)	VRPQQ	0 ± 1	0
148	1271	424 (2)	VRPQP	0 ± 0	0
149	1273	425 (1)	CGRTD	0 ± 0	0
150	1281	427 (3)	GAAAY	0 ± 0	0
151	1282	428 (1)	CGRNG	0 ± 1	0
152	1283	428 (2)	VRPHG	0 ± 0	0
153	1302	434 (3)	DAAAA	0 ± 0	0
154	1307	436 (2)	LRPQL	0 ± 0	0
155	1308	436 (3)	FAAAL	0 ± 1	0
156	1312	438 (1)	CGRIL	0 ± 0	0
157	1314	438 (3)	NAAAL	0 ± 0	0
158	1326	442 (3)	DAAAA	0 ± 1	0
159	1327	443 (1)	CGRTE	0 ± 0	0
160	1335	445 (3)	AAAAV	0 ± 0	0
161	1350	450 (3)	SAAAA	0 ± 1	0
162	1378	460 (1)	CGRSG	32 ± 14	14
163	1380	460 (3)	GAAAG	36 ± 5	16
164	1381	461 (1)	CGRRG	15 ± 0	6
165	1385	462 (2)	LRPHK	19 ± 7	8
166	1391	464 (2)	LRPHS	1 ± 1	0
167	1392	464 (3)	FAAAS	1 ± 1	0
168	1395	465 (3)	DAAAF	0 ± 0	0
169	1402	468 (1)	CGRNV	0 ± 0	0
170	1420	474 (1)	CGRNA	360 ± 26	154
171	1423	475 (1)	CGRTR	356 ± 8	152
172	1424	475 (2)	MRPHR	374 ± 4	160
173	1428	476 (3)	IAAAT	343 ± 15	147
174	1434	478 (3)	RAAAV	352 ± 17	151
175	1435	479 (1)	CGRMR	461 ± 19	197

176	1443	481 (3)	TAAAP	8 ± 3	3
177	1447	483 (1)	CGRTE	0 ± 0	0
178	1466	489 (2)	LRPQL	0 ± 0	0
179	1469	490 (2)	LRPQL	0 ± 0	0
180	1475	492 (2)	VRPQP	0 ± 0	0
181	1480	494 (1)	CGRTE	0 ± 0	0
182	1485	495 (3)	VAAAA	0 ± 0	0
183	1515	505 (3)	LAAAT	0 ± 0	0
184	1566	522 (3)	AAAAM	0 ± 1	0
185	1569	523 (3)	FAAAA	0 ± 0	0
186	1581	527 (3)	GAAAA	55 ± 14	24
187	1582	528 (1)	CGRTG	173 ± 17	74
188	1587	529 (3)	NAAAV	18 ± 2	8
189	1602	534 (3)	SAAAV	0 ± 0	0
190	1603	535 (1)	CGRIR	0 ± 0	0
191	1616	539 (2)	LRPQQ	0 ± 0	0
192	1622	541 (2)	LRPQL	0 ± 0	0
193	1626	542 (3)	SAAAH	0 ± 0	0
194	1627	543 (1)	CGRNS	0 ± 1	0
195	1628	543 (2)	MRPHS	0 ± 0	0
196	1639	547 (1)	CGRNC	0 ± 1	0
197	1665	555 (3)	CAAAI	0 ± 0	0
198	1682	561 (2)	VRPHL	0 ± 0	0
199	1708	570 (1)	CGRTA	0 ± 0	0
200	1710	570 (3)	AAAAA	0 ± 0	0
201	1717	573 (1)	CGRSR	0 ± 0	0
202	1718	573 (2)	VRPHR	0 ± 0	0
203	1721	574 (2)	LRPHE	0 ± 0	0
204	1729	577 (1)	CGRSR	0 ± 1	0
205	1767	589 (3)	IAAAA	0 ± 0	0
206	1770	590 (3)	NAAAI	0 ± 0	0
207	1776	592 (3)	HAAAA	0 ± 0	0
208	1796	599 (2)	VRPQL	0 ± 0	0
209	1797	599 (3)	DAAAL	0 ± 0	0
210	1808	603 (2)	LRPHE	0 ± 0	0
211	1811	604 (2)	LRPQL	84 ± 25	36
212	1821	607 (3)	IAAAQ	93 ± 12	40
213	1844	615 (2)	LRPQE	44 ± 2	19
214	1867	623 (1)	CGRIR	280 ± 14	103
215	1874	625 (2)	VRPHS	268 ± 24	114
216	1875	625 (3)	GAAAS	272 ± 19	116
217	1884	628 (3)	DAAAT	231 ± 9	99
218	1893	631 (3)	NAAAV	273 ± 16	117
219	1918	640 (1)	CGRKY	211 ± 8	90
220	1932	644 (3)	DAAAR	285 ± 6	122

221	1933	645 (1)	CGRSD	206 ± 13	88
222	1943	648 (2)	MRPHL	187 ± 4	80
223	1951	651 (1)	CGRNS	223 ± 19	95
224	1952	651 (2)	LRPHS	241 ± 18	103
225	1954	652 (1)	CGRTL	204 ± 7	87
226	1955	652 (2)	VRPQL	215 ± 14	92
227	1963	655 (1)	CGRIL	242 ± 7	104
228	1965	655 (3)	DAAAL	262 ± 4	112
229	1969	657 (1)	CGRKQ	256 ± 22	109
230	1970	657 (2)	MRPQQ	240 ± 5	102
231	1971	657 (3)	NAAAQ	284 ± 3	121
232	1974	658 (3)	SAAAN	298 ± 31	127
233	1975	659 (1)	CGRNR	294 ± 55	125

[a]Initially, DNA from 331 plasmid clones was isolated and sequenced to identify insertion sites. Plasmid clones containing the insertion outside the *MuA* ORF (6) and those yielding low quality sequence reads (12) were not included in the table. Several clones were isolated more than once. In these cases, only one representative clone was analyzed.

[b]Papillation analysis was performed by incubation at 30°C for 115 h on standard papillation medium (LB, Ap 100 µg/ml, Cm 20 µg/ml, Xgal 40 µg/ml, lactose 0.05 %, arabinose 0.1%). The data in this column indicate the means and standard deviations (SD) of papillae enumeration from the analyses of three colonies for each data point. The data for wild type MuA are 234 ± 22.
